# Carcinoembryonic antigen carrying SLe^X^ as a new biomarker of more aggressive gastric carcinomas

**DOI:** 10.7150/thno.33858

**Published:** 2019-10-11

**Authors:** Catarina Gomes, Andreia Almeida, Ana Barreira, Juliana Calheiros, Filipe Pinto, Rafaela Abrantes, Ana Costa, António Polonia, Diana Campos, Hugo Osório, Hugo Sousa, João Pinto-de-Sousa, Daniel Kolarich, Celso A. Reis

**Affiliations:** 1 Instituto de Investigação e Inovação em Saúde (I3S), Universidade do Porto, Porto, Portugal; 2 Institute of Molecular Pathology and Immunology of the University of Porto (IPATIMUP), Porto, Portugal; 3 Max Planck Institute of Colloids and Interfaces, Potsdam, Germany; 4 Institute for Glycomics, Griffith University, Southport, QLD, Australia; 5 Faculty of Medicine, University of Porto, Porto, Portugal; 6 Institute of Biomedical Sciences of Abel Salazar, ICBAS, Porto, Portugal

**Keywords:** Gastric Cancer, Carcinoembryonic antigen, Glycosylation, ST3Gal IV, Sialyl-Lewis X

## Abstract

Malignant transformation of gastric cells is accompanied by the deregulated expression of glycosyltransferases leading to the biosynthesis of tumor-associated glycans such as the sialyl-Lewis X antigen (SLe^x^). SLe^x^ presence on cell surface glycoconjugates increases the invasive capacity of gastric cancer cells and is associated with tumor metastasis. ST3Gal IV enzyme is involved in the synthesis of SLe^x^ antigen and overexpressed in gastric carcinomas. Herein, we identified the glycoproteins carrying SLe^x^ in gastric cancer cells overexpressing ST3Gal IV enzyme and evaluated their biomarker potential for gastric carcinoma.

**Methods:** SLe^x^ modified glycoproteins were identified applying western blot and mass spectrometry. Immunoprecipitation, proximity ligation assay (PLA), E-selectin binding assay and CRISPR/cas9 knockout experiments were performed to characterize the presence of SLe^x^ on the identified glycoprotein. Protein *N*-glycans of the SLe^x^ protein carrier were in deep analyzed by porous-graphitized-carbon liquid-chromatography and tandem mass spectrometry glycomics. *In silico* expression analysis of α2-3 sialyltransferase ST3Gal IV and SLe^x^ protein carrier was performed and the conjoint expression of the SLe^x^ modified glycoproteins evaluated by immunohistochemistry and PLA in a series of gastric carcinomas.

**Results:** Carcinoembryonic antigen (CEA; CEACAM5) was identified and validated by different methodologies as a major carrier of SLe^x^. *N*-glycomics of CEA revealed that complex *N*-glycans are capped with α2-3 linked sialic acid (Neu5Acα2-3Galβ1-4GlcNAc). Data set analysis of ST3Gal IV and CEA showed that ST3Gal IV expression was associated with patient´s poor survival, whereas CEA did not show any prognostic value. The co-expression of both CEA and SLe^X^ was observed in 86,3% of gastric carcinoma cases and 74,5% of the total cases displayed the conjoint CEA+SLe^x^ *in situ* PLA expression. This expression was associated with clinicopathological features of the tumors, including infiltrative pattern of tumor growth, presence of venous invasion and patient's poor survival. CEA immunoprecipitation from gastric carcinoma tissues also confirmed the presence of SLe^x^.

**Conclusion:** CEA is the major glycoprotein carrying SLe^x^ in gastric carcinoma and the conjoint detection of CEA-SLe^x^ is associated with aggressive tumor features highlighting its PLA detection as a biomarker of gastric cancer patient prognosis for theranostic applications.

## Introduction

Gastric cancer (GC) is one of the top five most frequently occurring cancers worldwide with poor survival and high death rate 1. The lack of specific early stage disease molecular markers urgently calls for a better understanding of the molecular mechanisms underlying gastric carcinogenesis and cancer progression. Altered glycosylation is a common feature in gastric carcinoma 2, 3. Glycosylation changes have been shown to be important in the gastric carcinogenesis process in which *Helicobacter pylori* adhesins recognize host gastric mucosa glycans and modulate its glycorepertoire to sustain a chronic infection 4-6. During gastric cancer progression, gastric carcinoma cells exhibit aberrant glycosylation on key proteins, such as integrins and E-cadherin. These alterations occurring on key glycoproteins are known to disrupt cell-extracellular matrix and cell-cell interactions leading to an invasive cancer phenotype 2, 3, 7.

Gastric cancer cell glycosylation is characterized by increased levels of terminal sialylated glycans, such as sialyl-Lewis X (SLe^x^; Neu5Acα2-3Galβ1-4[Fucα1-3]GlcNAc-R) that has been shown to correlate with an aggressive tumor phenotype and worse patient prognosis 8-10. SLe^x^, normally expressed at the cell surface of leucocytes, is known to be the major player during inflammation being essential in rolling and extravasation processes 11. In cancer cells, SLe^x^ expression is known to mimic this process determining invasion and development of metastases. The expression of the tumor-associated SLe^x^ antigen in gastric cancer cells is caused by the deregulation of key enzymes such as sialyl- and fucosyltransferases 12. SLe^x^ biosynthesis requires a sequential addition of α2-3 linked sialic acid onto *N*- and/or *O*-glycans by α2-3 sialyltransferase IV (ST3Gal IV) 12, 13 followed by insertion of a α1-3 linked fucose to the *N*-acetylglucosamine by the action of α1-3 fucosyltransferases 12, 14, 15. Gastric cancer cells exhibit an increased expression of the ST3Gal IV enzyme, which has also been correlated with aggressive tumors 16, 17. Indeed, in a previous work we demonstrated that the expression of ST3Gal IV, with no alterations in α1,3-fucosyltransferases expression, induced the expression of SLe^x^ in MKN45 gastric carcinoma cell line 12. In addition, this expression lead to the activation of signaling pathways, such as c-met and GTPases, involved in tumor cell invasive capacity 17. Here, we analyzed a glycoengineered gastric carcinoma cell line model that expresses SLe^x^ due to the overexpression of ST3Gal IV 12, in order to identify SLe^x^ protein carriers as novel biomarkers in gastric cancer.

Although association between glycan alterations in tumor tissues and clinical prognosis has been documented, its application in the clinical setting has been limited to few serological assays detecting circulating glycoconjugates shed by the tumors. Among these, the serological assays that have been approved are carcinoembryonic antigen (CEA) for colorectal cancer, cancer antigen 125 (CA125) for ovarian cancer, CA19.9 for pancreatic and gastric cancer, and prostate-specific antigen (PSA) for prostate cancer 18-20. Currently, the clinical application of these makers is mostly for monitoring treatment and relapses with no consensual application in patient's prognosis. This limited clinical application is mostly due to their low tumor specificity and sensitivity 19.

In the present work, we identified carcinoembryonic antigen (CEA, CEACAM5) as the major SLe^x^ protein carrier in gastric cancer cells. The presence of this glycoform was further demonstrated in gastric carcinoma tissues and associated with clinicopathological characteristics of the tumors. This finding demonstrates that the aberrant glycosylated CEA glycoform is associated with aggressive features of the tumors and highlights its potential as prognostic biomarker in gastric carcinoma and open windows for new therapeutic interventions.

## Materials and Methods

### Cell culture

MKN45 gastric carcinoma cell line (Japanese Cancer Research Bank, Tsukuba, Japan) was stably transfected with a full-length human gene for ST3GAL IV (MST3Gal IV) and the empty vector (Mock) and routinely grown as previously described 12.

### SDS-PAGE and western blot analysis

Cells at high confluence were incubated with NP40 lysis buffer and scrapped to obtain total protein cell lysates. Protein concentration was determined by the bicinchoninic acid protein assay (BCA)(Pierce), and protein extracts were loaded onto 7.5% sodium dodecyl-polyacrylamide gels for electrophoretic separation (SDS-PAGE) (Bio-Rad). Gel staining was performed by periodic acid-Schiff (PAS) method (Pierce) that specifically visualizes glycosylated proteins and images were acquired with a GS800 scanner (Bio-Rad). For blotting experiments gels were transferred onto nitrocellulose membranes and blocked with 5% bovine serum albumin (BSA) in phosphate-buffered saline (PBS) containing 0.05% Tween 20 (PBST). Western blotting was performed by incubating with primary antibodies followed by incubation with horseradish peroxidase-conjugated goat anti-mouse IgM or goat anti-mouse IgG1 secondary antibodies. For lectin blots, membranes were incubated with biotinylated SNA, MALI and MALII lectins followed by avidin/biotin complex (Vectastain) incubation (all from Vector Laboratories). Detection was performed by enhanced chemiluminescence (ECL) and film sheets exposure (both from GE Healthcare).

The following antibody and lectin dilutions were used: Anti-SLe^x^ clone KM93 at 1:500 (Millipore), Anti-SLe^x^ clone CSLEX1 at 1:1000 (BD biosciences), anti-CEA clone CB30 at 1:3000 (Cell Signaling Technology) and biotinylated SNA 1:600, biotinylated MALI 1:600, biotinylated MALII 1:600, horseradish peroxidase-conjugated goat anti-mouse IgM at 1:10,000 and goat anti-mouse IgG1 at 1:25,000 (Jackson Immunoresearch).

### Protein selection and identification by MALDI-TOF/TOF mass spectrometry

The bands highlighted on the western blot for SLe^x^ antigen were matched on the stained gel and proteins excised with a spotpicker (OneTouch 2D gel spotpicker, 1.5 mm diameter, Gel Company). After reduction and alkylation, the selected protein bands were *in-gel* digested with trypsin and the respective peptide mass spectra acquired by Matrix assisted laser desorption/ionisation - time of flight (MALDI-TOF/TOF) (4700 Proteomics Analyzer MALDI-TOF/TOF, AB SCIEX) as previously described 21. Proteins were identified using the combined information of Peptide Mass Fingerprint (PMF) and MS/MS peptide sequencing approaches by the Mascot protein search software (version 2.1.04, Matrix Science, UK), integrated in the GPS Explorer software (version 2.6, SCIEX, Framingham, MA). Protein searches were performed in the Swiss-Prot/UniProt protein database for the taxonomic selection *Homo sapiens*. The MS tolerance was 50 ppm for PMF analysis and 1.0 Da for MS/MS analysis. Cysteine carbamidomethylation and methionine oxidation were considered as constant and variable modifications, respectively. Protein scores greater than 56 (mowse score) were considered significant (p<0.05).

### CEA immunoprecipitation

ProteinG sepharose beads (60 µL) (Sigma) were incubated for 2 hours at 4 °C with 2 µL of anti-CEA antibody (Cell Signaling Technology), followed by antibody crosslinking using bis(sulfosuccinimidyl)suberate (Sigma). Cell line (600 µg) and tissue (200 µg) protein extracts were added to antibody ProteinG sepharose and incubated overnight at 4 °C. Beads were washed with 1% Triton X-100 PBS and eluted in Laemmli buffer for SDS-PAGE.

### PNGase F digestion

After CEA immunoprecipitation denaturating buffer was added and incubated at 100 °C for 10 min and samples were then digested ON with 1 unit of PNGase F according to the manufacturer's instructions (New England Biolabs) at 37 °C. PNGase F is an amidase that cleaves between the innermost GlcNAc and asparagine residues of high-mannose, hybrid and complex oligosaccharides from N-linked glycoproteins. The deglycosylated proteins were loaded onto 7.5% SDS-PAGE and immunoblotting was performed.

### CRISPR/Cas9 knockout

CEA knockout was performed using CRISP/Cas9 technology as described previously 22. Briefly, three different gRNAs (gRNA1: CATCTGTGGGGAGGGGCCGA; gRNA2: CAGGGGATGCACCATCTGTG and gRNA3: GATGCACCATCTGTGGGGAG) were designed using deskgen platform (https://www.deskgen.com/landing/#/) and gRNA2 selected for plasmid production (Figure S1). After cell transfection, single cell sorting was performed to obtain different clones. Two clones were selected from both cell lines (Mock and MST3Gal IV) for the following experiments.

### E-selectin binding assay

Cell lysates were collected using selectin chimera lysis buffer (150 mM NaCl, 2 mM CaCl_2_, 50 mM Tris, pH 7.4, 20 µg mL^-1^ PMSF, complete protease Inhibitor Cocktail EDTA-free and 2% NP40), and 500 µg of total protein extracts pre-cleared for 1 h at 4 °C using 30 µL of ProteinG sepharose beads (GE healthcare). Pre-cleared protein extracts were incubated with 3 µg of Fc-chimera E-selectin (R&D) for 2 h at 4 °C prior overnight incubation with 60 µg of ProteinG sepharose beads previously blocked with 1% BSA selectin chimera lysis buffer. Beads were washed with selectin chimera lysis buffer. Proteins were eluted in Laemmli buffer and separated by SDS-PAGE for further western blot analysis.

### Immunofluorescence staining

Cells were seeded and cultured on glass coverslips in 24-well plates (Orange Scientific), washed with PBS and fixed with methanol. Blocking was performed using swine or rabbit serum in 10% BSA PBS at room temperature followed by primary antibody incubation overnight at 4 °C. Secondary antibodies were incubated and nuclear counter staining was done using DAPI. Washes were performed with PBS. Fluorescence signal was examined in a fluorescence microscope and images were acquired using a Zeiss Axio cam MRm and the AxioVision Rel. 4.8 software (Carl ZEISS).

Monoclonal antibodies were used in the following dilutions: Anti-SLe^x^ clone KM93 at 1:60 (Millipore), anti-CEA clone CB30 at 1:600 (Cell Signaling Technology); secondary antibodies: rabbit anti-mouse 1:100 and swine anti-rabbit 1:70 FITC-conjugated (DAKO).

### Release of *N*-glycans and PGC-nanoLC-ESI-MS/MS analysis

The *N*-glycans of immunoprecipitated CEA (less than 500 ng) were released from PVDF membrane immobilized glycoproteins as previously described 23. The released *N*‑glycans were analyzed by PGC (porous graphitized carbon) nanoLC-ESI-MS/MS. Glycans were manually annotated as described by Hinneburg *et al*. using the GlycoMod tool available on the ExPASy server (http://web.expasy.org/glycomod/) and Glycoworkbench software 2.1 24-26.

### Data processing and relative *N*-glycan quantitation

The peak area of each glycan composition and isoform was calculated by the respective extracted ion chromatograms using Compass QuantAnalysis (Bruker, Bremen). The generated data was imported in CSV format to R and expressed as a relative abundance value for each individual *N*-glycan structure, respectively, within each cell line. All identified CEA* N*-glycans are listed in Supplementary Table 1 and monosaccharides are represented following the recommendations of the SNFG 27.

### Tissue samples and immunohistochemistry analysis

Gastric carcinomas and gastric mucosa adjacent to carcinomas were obtained from individuals undergoing surgery at Centro Hospitalar São João (CHSJ), University of Porto Medical Faculty (Porto, Portugal). The study was performed with the approval of the local CHSJ ethical committee. Formalin-fixed paraffin-embedded gastric carcinoma tissues (n=51) were used for slide sections and stained with hematoxilin-eosin for histological examination. Hematoxylin and eosin-stained sections were used to classify gastric carcinomas according to Carneiro *et al.* (1995)^28^, Laurén (1965)^29^ and Ming (1977)^30^. Clinicopathological characteristics of the cases (lymphatic invasion, venous invasion and pTNM staging) were also recorded for every case. Immunoreactivity was classified into 4 categories based on % of positive carcinoma cells (neg=0%; <25%; 25%-75%; and >75%). Frozen tissues from aggressive gastric carcinoma patients (n=8) and from patients that underwent a gastric bypass (n=2) were used for protein extraction for further western blot analysis. Prior protein extraction, tissues were washed three times with fresh PBS. Protein extraction was performed using RIPA buffer (in the proportion of 750 µL per 100 mg of tissue) containing Ditiotreitol (DTT) and protease cocktail inhibitors and a pestle mixer was used for tissue homogenization. Ultimately, tissues were sonicated for 5 min at medium intensity and samples centrifuged at 12,000 g for 15 min. Supernatant were collected and proteins concentration determined by the BCA assay (Pierce).

For immunohistochemistry paraffin sections were dewaxed, rehydrated and blocked for endogenous peroxidase activity. Sections were then incubated with normal rabbit serum followed by incubation with the monoclonal antibodies overnight at 4 °C. A secondary biotinylated rabbit anti-mouse (DAKO) antibody was used followed by avidin/biotin complex (Vectastain) incubation. Detection was performed using 3,3′-diaminobenzidine tetrahydrochloride (DAB) (Sigma) containing 0.02% hydrogen peroxide and counter staining of the nucleus was done with Mayer's hematoxylin.

Mouse monoclonal antibodies were used in the following dilutions: Anti-SLe^x^ clone KM93 at 1:60 (Millipore), anti-CEA clone CB30 at 1:300 (Cell Signaling Tecnhnology) after antigen retrieval with citrate buffer (10 mM Citric Acid, pH 6.0).

### Proximity ligation assay (PLA)

PLA assay was performed in cells cultured on glass coverslips and on gastric carcinoma tissue sections. According to previous studies, PLA is an appropriate approach for protein-glycan interaction detection in tissues 31. Thus, we used this approach to evaluate the presence of SLe^x^ on CEA in tissue sections. Briefly, paraffin tissue sections were dewaxed and rehydrated followed by antigen retrieval with citrate buffer. Tissue sections and cells on glass coverslips were then incubated with normal goat serum diluted 1:5 in 10% BSA PBS followed by incubation with a solution containing the two monoclonal antibodies overnight at 4 °C. PLA probes anti-IgG plus and anti-IgM minus were incubated 1 h at 37 °C. PLA reactions were performed using the DuoLink® II Fluorescence Kit (Olink® Bioscience) according to the manufacturer's instructions. Nuclei were stained with DAPI and slides were mounted in an appropriated medium (Duolink II). PLA products were seen as fluorescent red dots. Fluorescence was examined in a fluorescence microscopy and images were acquired using a Zeiss Axio cam MRm and the AxioVision Rel. 4.8 software (Carl ZEISS).

### *In silico* survival analysis in human patient samples

*In silico* survival data were obtained from the 2018 version of the Kaplan-Meier (KM) Plotter gastric cancer survival database (http://kmplot.com/analysis/index.php?p=service&cancer=gastric) 32, 33. The database compromises a total of 1065 samples (GSE14210, GSE15459, GSE22377, GSE29272, GSE51105, and GSE62254) with survival data for 882 GC patients. The clinical relevance of *ST3GAL IV* (203759_at) and *CEACAM5* (201884_at) expression was analyzed by KM survival plots. Patients were stratified in two groups by the median expression for each probe analyzed (high group > median expression, and low group < median expression). Overall survival was analyzed for all gastric cancer patients and then divided by stage (I n = 69; II n = 145; III n = 319; and IV n = 152) and Lauren subtype classification (intestinal n = 336; diffuse n = 248; and mixed n = 33). The hazard ratio (HR) with 95% confidence interval and log-rank *p-*values were calculated by KM plotter software.

### Statistics

All statistical analyses were performed using SPSS Software. The unpaired t-test was used to determine significant differences. To determine the overall survival of gastric cancer patients CEA+SLe^x^ positive vs negative, Kaplan-Meier analyses was used and the log-rank Mantel-Cox test was employed to determine any statistical difference between the survival curves of the cohorts. Statistical significance was set at the alpha level = 0.05.

## Results

### Identification of altered sialylated glycoprotein in ST3Gal IV expressing gastric carcinoma cells

An increase in the SLe^x^ antigen (NeuAcα2-3Galβ1-4[Fucα1-3]GlcNAc-R) expression has been shown to be induced by the expression of the ST3Gal IV sialyltransferase 12, 13, 16, 34. Previously, we showed that the expression of ST3Gal IV in gastric carcinoma cells leads to SLe^x^ biosynthesis on both membrane-associated and secreted glycoproteins 12, 17 with a specific range of glycomic changes characterized mainly by the increased expression of α2-3 linked *N*‑acetylneuraminic acid (NeuAc) 17, 35.

**Figure 1:**
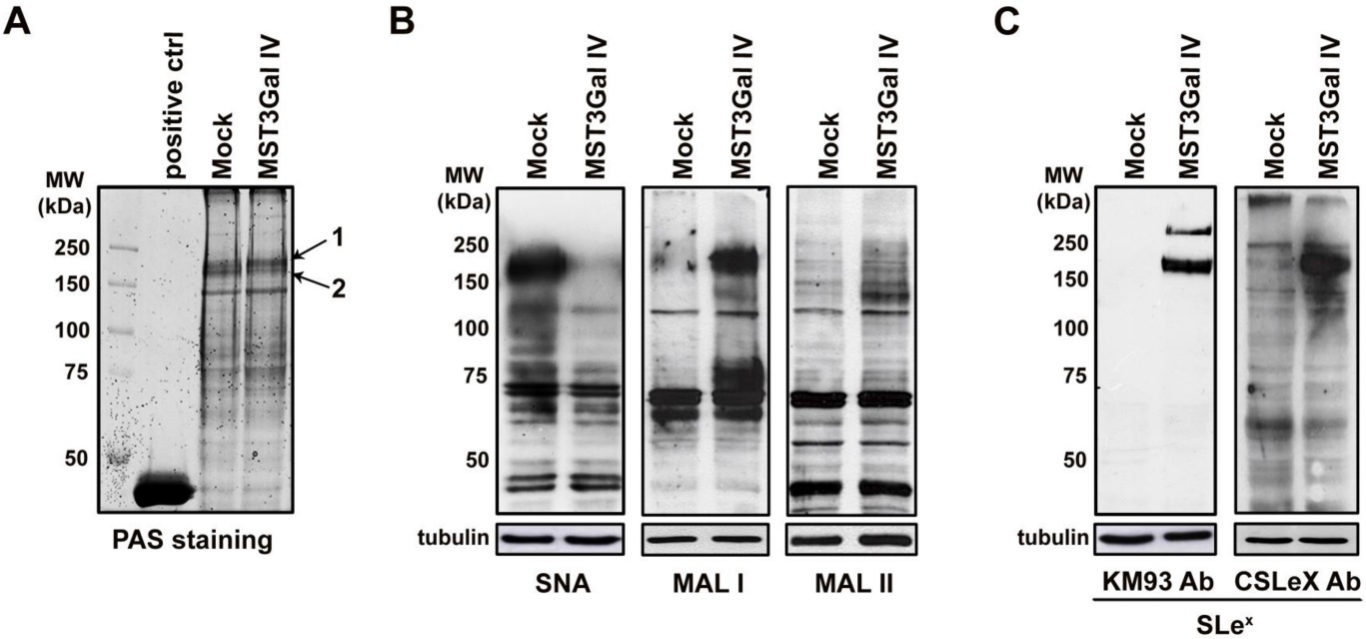
** Switching of protein sialylation in ST3Gal IV expressing gastric carcinoma cells leading to SLe^x^ expression. A -** Periodic Acid-Schiff (PAS) staining of SDS-PAGE glycoproteins from Mock and MST3Gal IV total cell lysates showed no differences in total cell protein glycosylation on both cell lines; Horseradish peroxidase was used as positive staining control; Arrows 1 and 2 indicate the bands selected for protein identification by MALDI-TOF/TOF mass spectrometry. **B -** Evaluation of α2-6 and α2-3 linked NeuAc by SNA, MAL I and MAL II lectin staining on Mock and MST3Gal IV cells. Results showed a reduce intensity of α2-6 linked NeuAc carrying glycans accompanied by an increase in α2-3 linked NeuAc at high molecular weight protein in ST3Gal IV expressing cells. Mackia ammorensis lectins, capable of recognizing different α2-3 linked NeuAc carrying glycans structures, further demonstrated the presence of α2-3 linked NeuAc in Galβ1-4GlcNAc structures; **C -** Western blot analysis of SLe^X^ structures using two different antibodies showed the presence of SLe^x^ antigens on high molecular weight proteins from ST3Gal IV expressing cells with no expression on Mock control cells.

The evaluation of total protein cell lysates from ST3Gal IV expressing and Mock control cells stained with PAS showed that the expression of glycosylated proteins was similar in both cell lines, demonstrating that ST3Gal IV enzyme expression did not affect the total amount of glycoproteins (Figure 1A). The evaluation of α2-3 and α2-6 linked NeuAc by lectin far-western blotting analysis showed that α2-6 sialylation of high molecular weight proteins is reduced in ST3Gal IV transfected cells whereas α2-3 sialylation is increased (Figure 1B). According to MAL preferred binding substrates 36, we observed that α2-3 NeuAc is apparently linked to Galβ1-4GlcNAc structures (MAL I results). Additionally, SLe^x^ staining using to different monoclonal antibodies was only observed at high molecular weight glycoproteins expressed in MST3Gal IV cells (Figure 1C) with no SLe^x^ staining on Mock control cells. These data indicated a sialylation switching towards α2-3 linked NeuAc specifically at high molecular weight proteins in ST3Gal IV transfected cells, being SLe^x^ a major antigen detected.

### CEA is a major carrier of SLe^x^ in gastric carcinoma cells

The differential protein specific sialylation observed in ST3Gal IV expressing cells allowed the identification of protein(s) carrying these altered glycans. Match SLe^x^ positive bands (Figure 1A black arrows) were excised and identified using MALDI-TOF/TOF mass spectrometry, leading to the identification of two main proteins (Table S1). Carcinoembryonic antigen (CEACAM5; CEA) was identified in both gel bands 1 and 2 while Ras GTPase-activating-like protein IQGAP, which according to the uniprot database and scientific literature is not glycosylated, was found as a contaminant in band 2 (Table S1). These results show that there is an inherent specificity to add SLe^x^ onto specific protein carriers and CEA is possibly the major target.

**Figure 2:**
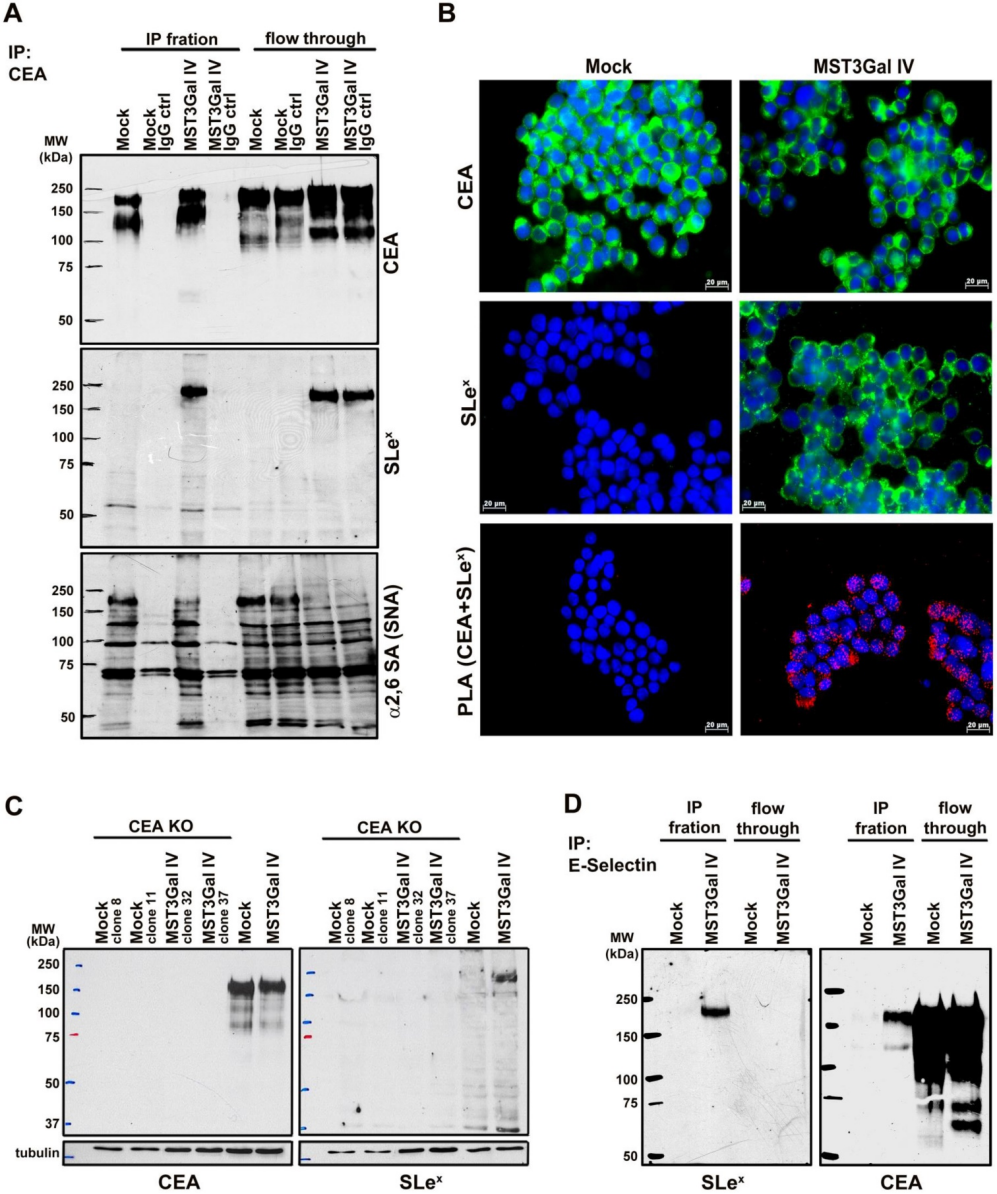
** CEA is the carrier of SLe^x^ in MST3Gal IV cell line.** Four independent assays were employed to assess the presence SLe^x^ on CEA in Mock and MST3Gal IV cells: immunoprecipitation assay, PLA, CEA KO by CRISPR/Cas9 and E-selectin immunoprecipitation. **A -** CEA immunoprecipitation in MST3Gal IV and Mock cell lysates. Upper panel: CEA western blot analysis confirmed the expression of CEA in Mock and MST3Gal IV cells; middle panel: in MST3Gal IV cells the SLe^x^ epitope is largely attached to CEA glycans; lower panel: CEA expressed in MST3Gal IV cells showed a reduced presence of α2-6 NeuAc on CEA high molecular weight glycoforms. Immunoprecipitation flow through and a mouse IgG immunoprecipitation assay were used as experiment control. **B -** Immunofluorescence analysis confirmed the presence of CEA in Mock and MST3Gal IV gastric carcinoma cells while SLe^x^ is just found in the MST3Gal IV overexpressing cell line. The positive PLA signal only observed in MST3Gal IV cells is indicative of the close proximity of CEA and SLe^x^. **C -** Knockout of CEA in both cell lines using CRISPR/Cas9 lead to the loss of SLe^x^ expression in MST3Gal IV cells.** D -** Immunoprecipitation of SLe^x^ carrying glycoproteins using E-selectin Fc protein chimera showed the high specificity of E-selectin for capturing SLe^x^ expressing glycoproteins in MST3Gal IV cells. A CEA western blot analysis after E-Selectin immunoaffinity enrichment was only positive for the MST3Gal IV cell lysates, clearly emphasizing the presence of SLe^X^ on CEA.

This was further confirmed by using CEA immunoprecipitation, followed by western blot analysis for SLe^x^ antigen and α2-6 sialylation. Albeit the results confirmed that both cell lines expressed CEA (Figure 2A upper panel), only CEA from ST3Gal IV transfected cells carried SLe^x^ epitopes (Figure 2A middle panel). Additionally, SNA staining indicated a decrease of α2-6 sialylation in the same molecular weight region (Figure 2A lower panel). Interestingly, CEA immunoprecipitated from MST3Gal IV cells displayed a slightly higher molecular weight when compared to the one from Mock cells as determined by the reduced SDS-PAGE migration (Figure 2A upper panel). These differences in the SDS-PAGE migration behavior may reflect the altered glycosylation found in CEA from MST3Gal IV cells. In addition, the concurrent expression of SLe^x^ and α2-6 NeuAc suggests that the glycosyltransferases performing the terminal sialylation compete for the CEA acceptor substrate that was identified as the major glycoprotein modified with SLe^x^ after ST3Gal IV transfection in gastric cancer cells.

Additional confirmation of SLe^x^ antigen presence on CEA in gastric cancer cells was achieved by PLA. PLA positive signals were only observed for MST3Gal IV cells confirming the molecular proximity of SLe^x^ and CEA in this cell line, whereas no PLA signal was observed in the Mock control cells (Figure 2B). Further, CEA silencing by CRISPR/Cas9 knockout also leads to the loss of SLe^X^ expression in two MST3Gal IV cell clones, demonstrating that CEA is a major carrier of this glycan in ST3Gal IV transfected gastric cancer cells (Figure 2C)**.** An ortogonal approach to substantiate the presence of SLe^x^ was performed using the recombinant E-selectin binding assay. It is well established that SLe^x^ is a ligand for E-selectin 11. We performed an immunoprecipitation assay using a recombinant human E-selectin on both Mock and MST3Gal IV cell lysates followed by western blot analysis for SLe^x^ and CEA (Figure 2D). A positive band for SLe^x^ was observed exclusively in the E-selectin immunoprecipitation from MST3Gal IV cell lysates, demonstrating the presence of a glycoprotein bearing the SLe^x^, the ligand for E-selectin. Additionally, a band corresponding to CEA was also observed in the E-selectin immunoprecipitation from MST3Gal IV lysates supporting that CEA carries SLe^x^ structures.

### Structural demonstration of CEA α2-3 sialylation in ST3Gal IV expressing gastric carcinoma cells

**Figure 3:**
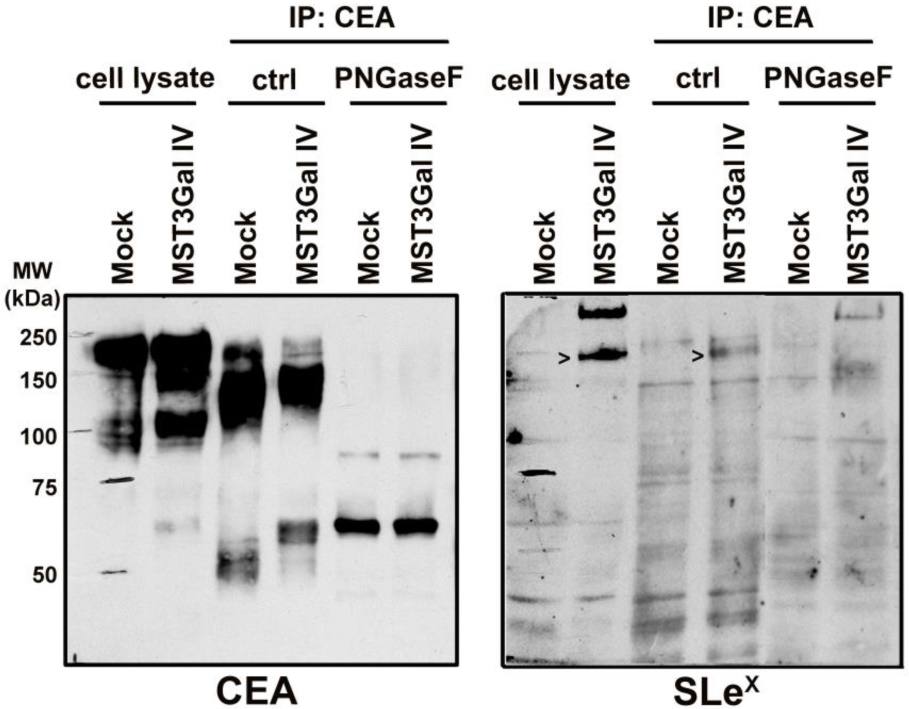
** PNGase F treatment on immunoprecipitated CEA demonstrated the presence of SLe^x^ in *N*-glycans.** CEA from both cell lines is heavily *N*-glycosylated as PNGase F mediated deglycosylation shifts the SDS-PAGE migration of CEA from 150-200 kDa towards its predicted molecular weight of around 71 kDa. The PNGase F treated CEA from MST3Gal IV cells was negative in the SLe^x^ western blot analysis confirming that the SLe^x^ epitope is present on CEA *N*-glycans (black arrows).

**Figure 4:**
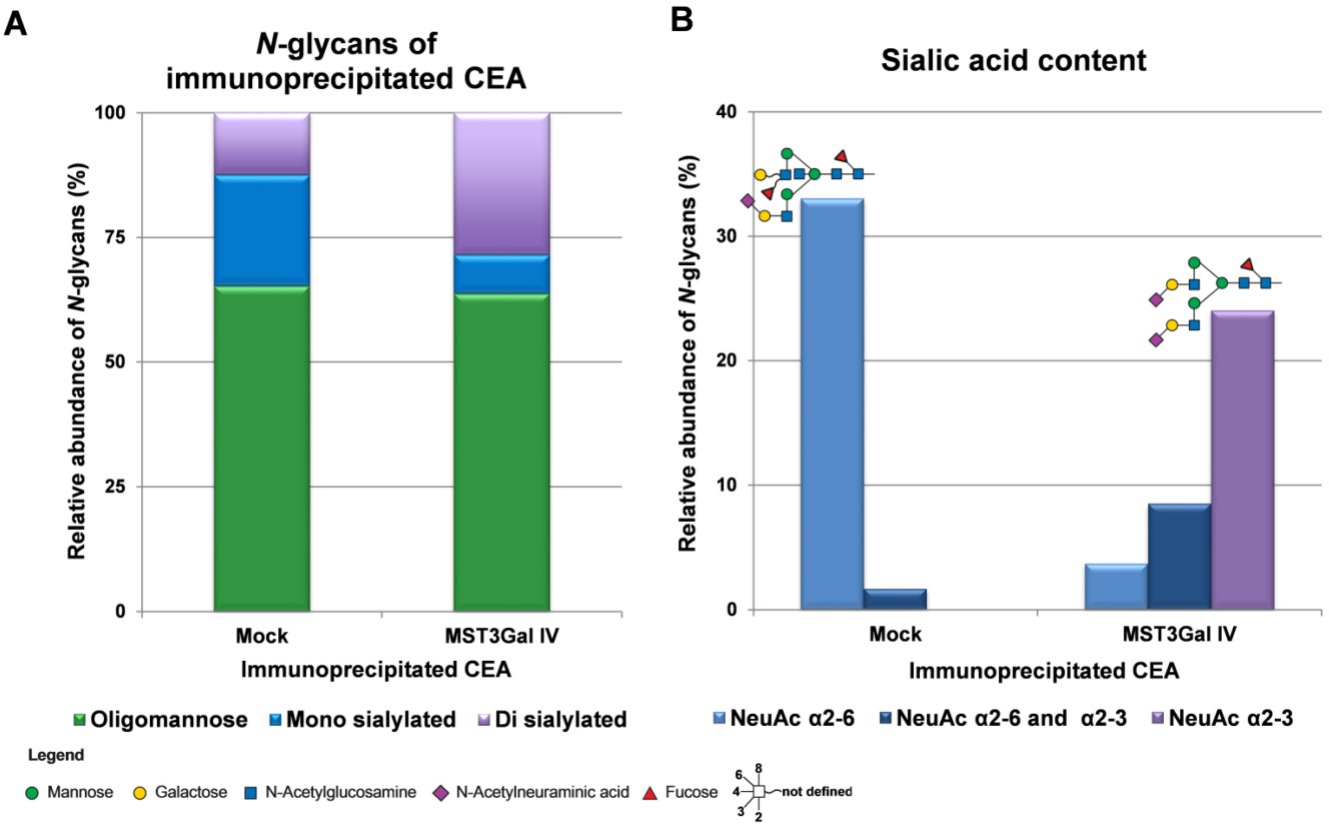
** Overview on the *N*-glycome of CEA immunoprecipitated from Mock and MST3Gal IV cells.** *N*-glycans were released from immunoprecipitated CEA, analyzed by porous graphitized carbon nanoLC-ESI-MS/MS and qualitatively and quantitatively assessed. The *N*‑glycans were classified into 3 classes: oligomannose (green), mono sialylated (blue) and di sialylated (purple). **A -** CEA immunoprecipitated from Mock and MST3Gal IV cells exhibited different complex *N*-glycan profiles despite similar levels of oligomannose *N*-glycans. Mock cells expressed CEA was mainly carrying monosialylated *N*-glycans while disialylated *N*-glycans were the major complex type *N*-glycans in MST3Gal IV overexpressing cells. **B -** Mock cell derived CEA carried type II *N*-glycans (Galβ1-4GlcNAc) capped with α2-6 linked NeuAc (light blue) while CEA from MST3Gal IV cell lines carried type II *N*-glycans capped with α2-3 linked NeuAc (purple). The major sialylated *N*-glycan structures of immunoprecipitated CEA were highlighted on top of each linkage type. The symbol nomenclature for glycans (SNFG) recommendations were followed for glycan structure depiction 27. Detailed information of the identified *N*-glycans is summarized in Table S2 and Figure S2.

CEA is a heavily glycosylated protein with 28 possible sites of *N*-glycosylation 37. The above-mentioned findings prompted us to explore the CEA glycan compositions in gastric cancer cells in more detail. The initial CEA-deglycosylation assay using PNGase F endoglycosidase to release all *N*-glycans showed a drastic increase of CEA SDS-PAGE migration, clearly demonstrating that *N*-glycans contribute to roughly half the size of the CEA glycoprotein (Figure 3). The band smear, observed around 200 kDa, was reduced to sharp bands detected at positions corresponding to approximately 80 and 60 kDa, respectively. Also, SLe^x^ detection on CEA molecules from MST3Gal IV cells was completely abolished by PNGase F treatment (Figure 3). We also performed detailed *N*-glycomics on immunoprecipitated CEA derived from both cell lines using our PGC nanoLC ESI-MS/MS approach 23. Albeit the abundance of oligomannose type *N*-glycans remained similar, the *N*-glycan profiles of CEA derived from Mock and MST3Gal IV cells showed distinct components in the complex type *N*-glycan distribution (Figure 4A). Oligomannose type *N*-glycans contributed around 60% of the total CEA *N*-glycan pool, whereas approximately 40% were sialylated *N*-glycans. Complex type *N*-glycans on CEA obtained from Mock cells carried mostly a single α2-6 linked NeuAc residue while the ones present on CEA from MST3Gal IV cells showed an increase in disialylated *N*-glycans and high levels of α2-3 sialylation (Figure 4B, Table S2 and Figure S2).

### ST3Gal IV expression is a marker of poor prognosis in gastric cancer patients

High expression levels of both CEA and SLe^x^ have been described in gastrointestinal tumors 8, 9, 38. Following up on our *in vitro* results, we sought to investigate whether the apparent CEA-SLe^x^ crosstalk could also be observed in gastric cancer (GC) patients.

**Figure 5:**
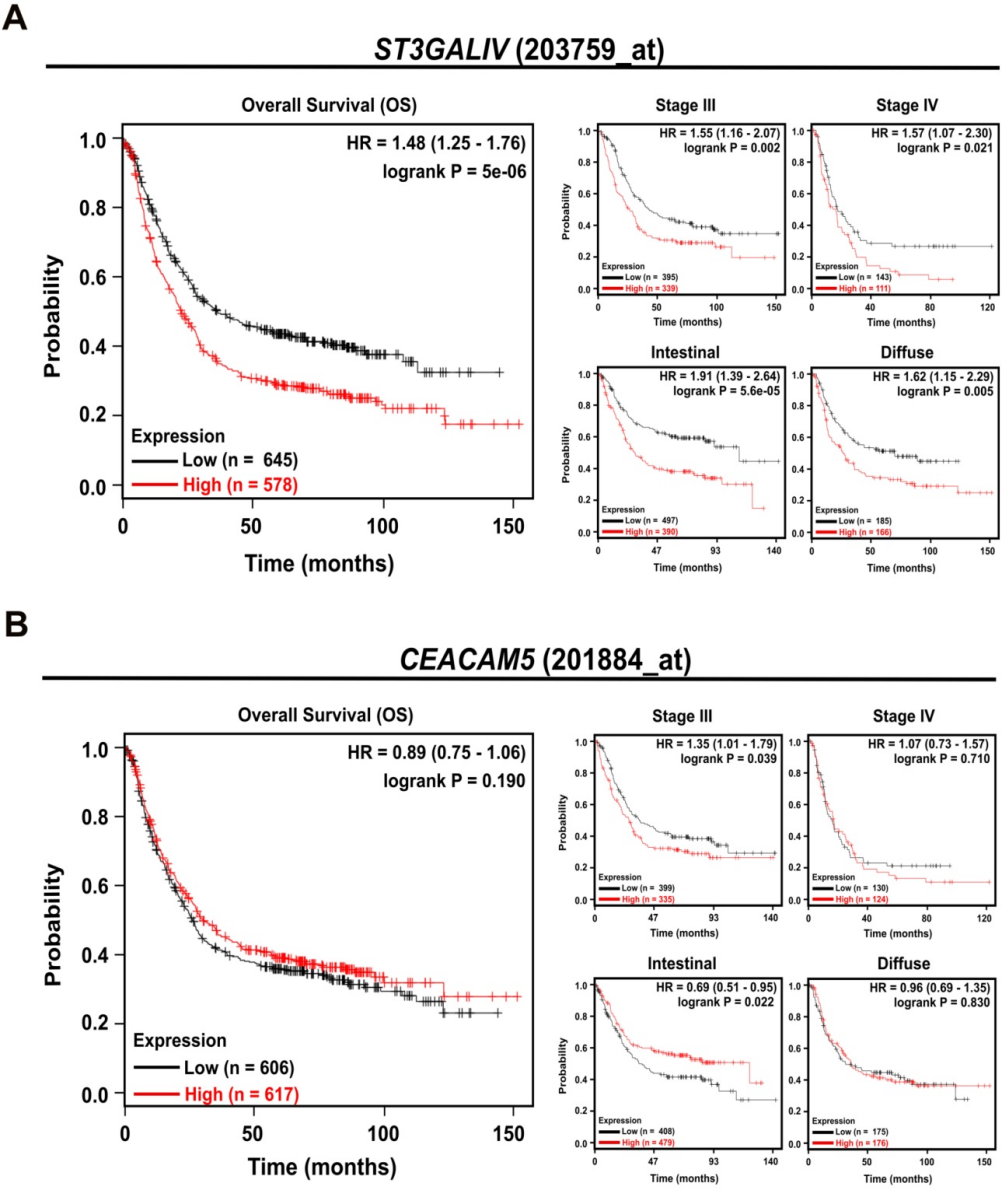
** ST3Gal IV expression predicts overall survival in gastric cancer patients.** A publicly available database (Kaplan-Meier Plotter) was used to investigate the effect of *ST3Gal IV* and *CEACAM5* expression on overall survival (OS) in a cohort of 882 gastric cancer patients. Patients were divided in two groups by the median expression for each probe used (*ST3Gal IV*: 203759_at; *CEACAM5*: 201884_at). **A -** High ST3Gal IV expression predicts a worse prognosis, whereas **B -** CEACAM5 did not have any effect on the OS of gastric cancer patients. *p-*value, hazard ratio (HR), and confidence interval (CI) were calculated by the log-rank method.

To determine whether ST3Gal IV and CEA (*CEACAM5*) expression have an impact on survival in GC patients, we extended our studies to a large independent, publicly available microarray data set of 882 patients (Kaplan-Meier Plotter 32 (http://www.kmplot.com/). We observed that patients with high ST3Gal IV expression levels exhibited a significantly reduced overall survival (OS), approximately 20%, compared to patients with low ST3Gal IV expression, approximately 40%, within 10 years (HR = 1.48, CI = 1.25-1.76, p < 0.001) (Figure 5A). This poor prognosis also correlated with GC stage aggressiveness (stage III: HR = 1.55, CI = 1.16-2.06, p = 0.002; and stage IV: HR = 1.57, CI = 1.07-2.30, p = 0.021) and appeared to be tumor type independent (intestinal: HR = 1.91, CI = 1.39-2.64, p < 0.001; diffuse: HR = 1.62, CI = 1.15-2.29, p = 0.005). The prognostic impact of *CEACAM5* expression alone, however, did not appear to have any reasonable prognostic value for these gastric cancer patients as essentially no association with the evaluated parameters was found. Only in patients diagnosed with an intestinal subtype a high *CEACAM5* expression appeared to support a favorable prognosis (HR = 0.69, CI = 0.51-0.95, p = 0.022, Figure 5B). This *in silico* results pointed to the importance of ST3Gal IV expression in gastric cancer progression and patients' poor prognosis.

Driven by the *in silico* analysis, we attempted to evaluate the expression of ST3Gal IV by immunohistochemistry in a series of gastric carcinoma patient's. Unfortunately, there was no available ST3Gal IV antibody that worked for immunohistochemistry analysis.

### α2-3 sialylation on CEA is a feature of more aggressive gastric carcinoma tissues

Motivated by these analyses we further investigated the conjoint presence of the SLex glycan structure with CEA in gastric carcinoma tissues and determine how well such a dual signature associates with clinicopathological features of the cases and patients' prognosis. We used immunohistochemistry to firstly evaluate the expression of both SLe^x^ and CEA in a series of 51 GC tissues (Table 1). Normal and adjacent mucosa showed no CEA expression while a few cells exhibited a weak immunostaining for SLe^x^ in gastric mucosa with inflammatory cell infiltration. The majority of GC tissues 50 (98.0%) showed CEA expression with staining intensity varying from less than 25% to more than 75% of the carcinoma cells. CEA expression showed to be associated with venous invasion. The SLe^x^ expression was observed in 44 (86.3%) out of 51 gastric carcinoma cases with intensity also varying from less than 25% to more than 75% of carcinoma cells (Table 1). The SLe^x^ expression levels were associated with cases showing an infiltrative pattern of growth (Ming´s classification).

**Figure 6:**
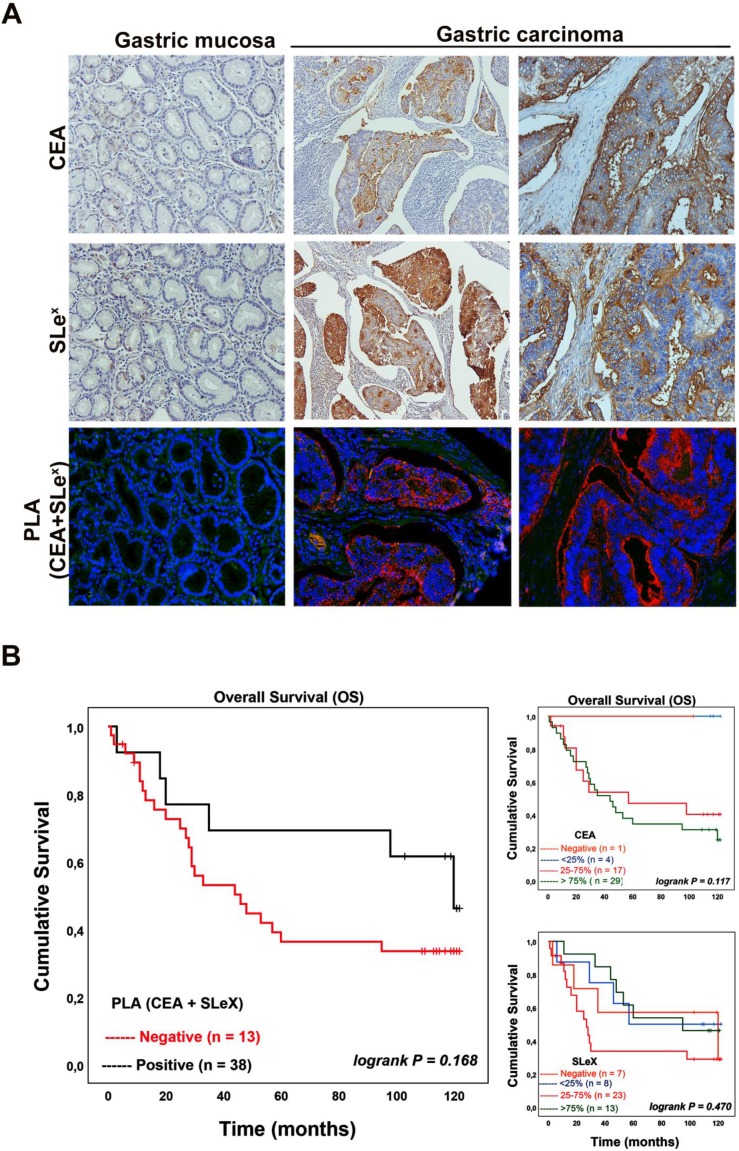
** CEA is a SLe^X^ carrier in gastric carcinoma tissues and is associated with poor patient´s overall survival. A -** Representative images of the immunohistochemical expression of CEA, SLe^x^ and PLA for both CEA+SLe^x^ in two gastric carcinoma cases as well as in normal gastric mucosa. Normal gastric mucosa (left column) was CEA and SLe^x^ negative, while two exemplary gastric carcinoma tissue samples (middle and right column) were positive for both epitopes. The PLA signal indicative for a conjoint presence of CEA-SLe^x^ was only observed in gastric carcinoma tissues (200x magnification). **B -** Kaplan-Meier analysis for CEA, SLe^X^ and the CEA+SLe^X^. CEA+SLe^X^ showed a strong tendency to be associated with poor patient's overall survival.

Further, PLA analysis was performed to confirm the conjoint presence of SLe^x^ antigen with CEA in gastric carcinoma tissues (Table 2). No signal was observed in normal or adjacent gastric mucosa as well as in SLe^x^ or CEA negative cases. In gastric carcinomas, 38 out of 51 cases (74.5%) gave a positive CEA+SLe^x^ PLA signal. Also, 38 out of the 44 SLe^x^ positive cases (displaying from 25-100% of positive cells) were positive in PLA, reinforcing the finding that CEA is a SLe^x^ carrier in gastric carcinoma. Interestingly, the negative cases in the PLA analysis were negative or present less than 25% SLe^x^ positive cancer cells. Figure 6A shows representative images of the immunohistochemical expression of CEA, SLe^x^ and PLA for both CEA+SLe^x^ in two gastric carcinoma cases as well as in normal gastric mucosa. In the analyzed gastric carcinoma cohort, the association between the PLA signal and clinicopathological variables showed a statistical significant correlation with advanced stage carcinomas (stage III+IV) (90.5% of cases, Table 2). In addition, there is a strong tendency to be associated with the presence of venous invasion (83.3%; *p*-value 0.081) and with infiltrative pattern of tumor growth (Ming classification) (83.9%; *p*-value 0.063). Notably, taking in consideration the conjoint expression of CEA+SLe^x^ with patient´s overall survival (Table 2), we observed that PLA positive cases display a strong tendency for a reduction in the overall patient's survival (Figure 6B).

**Figure 7:**
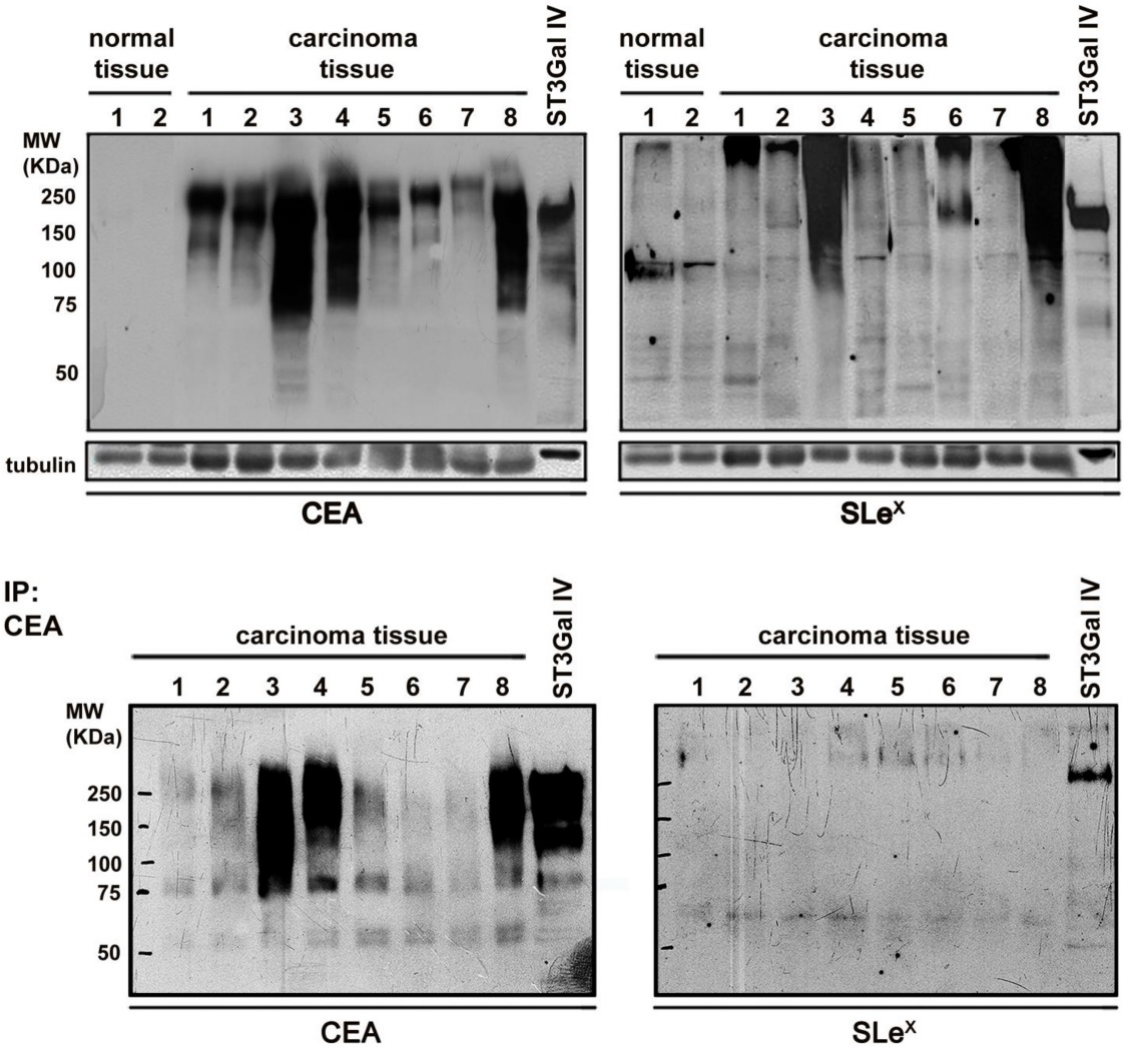
** Analysis of the conjoint presence of CEA+SLe^X^ in fresh human gastric carcinoma tissues** Western blot expression analysis of CEA and SLe^x^ in protein extracts from gastric carcinoma tissues and healthy controls (upper panel) and in immunoprecipitated CEA (down panel). CEA is present in all the gastric carcinoma tissues with no expression in healthy controls. SLe^X^ was present in 7 out of the 8 studied cases. CEA immunoprecipitation confirmed the presence of SLe^X^ epitopes in CEA glycoforms in some cases.

Further validation of the immunohistochemistry results was performed using protein extracts from eight frozen advanced gastric carcinoma tissues and two control samples followed by western blot analysis of CEA and SLe^x^ (Figure 7). All the gastric carcinoma cases were positive in the CEA western blot with no staining in the control samples. Interestingly, the gastric carcinoma samples exhibited a high molecular weight range of CEA molecules, indicating the presence of multiple CEA glycoforms. In addition, seven out of the eight analyzed cases also showed SLe^x^ carrying proteins. CEA immunoprecipitation followed by SLe^x^ western blot analysis confirmed that CEA is a SLe^x^ carrier (Figure 7).

## Discussion

Changes in the expression of sialylated glycans such as the SLe^x^ antigen are one of the most common cancer associated glycosylation alterations and have been associated with malignant behavior of cancer cells and more aggressive tumors 9, 39-41. In this work, we explored the importance of SLe^x^ antigen in gastric cancer to identify markers with potential theranostic applications. Following up on earlier studies from our group where the increased expression of ST3Gal IV induced SLe^x^ antigen expression generating an *in vitro* model of a more aggressive gastric carcinoma 17, the present study identified CEA to be the major protein carrier of SLe^x^. This *in vitro* finding was validated in a cohort of gastric carcinomas. Thus, the conjoint CEA+SLe^x^ presence in the gastric carcinoma cell line and in gastric carcinoma tissues uncovered a potential new prognostic gastric cancer biomarker that can be easily translated into a clinical setting and open the opportunity for the development of a new targeted therapy.

The relevance of tumorigenesis associated glycosylation changes has previously been described for cancer biomarkers used in the clinic. For instance, in benign prostate lesions, the prostate specific antigen (PSA) glycosylation pattern differs from the one present in malignant cases. Therefore, PSA distinct glycosylation constitutes a key component for the application of this serological biomarker feature in the clinics 42, 43. In the case of CEA, its overexpression has been well-documented in gastric cancer 44, 45 where increased serum CEA levels correlate with a poor disease outcome 46, 47. Nevertheless, there is a significant lack of information on CEA associated glycosylation alterations in gastric carcinogenesis. Previous reports already suggested that CEA glycosylation is altered in cancer tissues. For instance, CEA isolated from normal colon mucosa and pre-neoplastic lesions exhibited a different molecular weight compared to the one obtained from colon cancer cells 48. Recently, a lectin microarray platform demonstrated that CEA from colorectal carcinoma patient tissues have a distinct pattern of glycosylation compared to normal tissue 49 and lately it was identified *N*-glycan compositions indicative for the presence of outer arm fucose residues such as Lewis antigens 50. In the present study, we found that CEA is the carrier of the cancer associated antigen SLe^X^ in gastric carcinoma. Using a gastric carcinoma cell model that expresses SLe^X^ due to ST3Gal IV overexpression, we observed a reduced α2,6 sialylation accompanied by an increased α2,3 sialylation in specific glycoproteins. Using mass spectrometry analysis, we identify CEA as the major glycoprotein and lectin and antibody western blot characterization disclose SLe^x^ as the α2,3 sialylated structure. Further, the CEA *N*-glycan compositions from the gastric carcinoma cells were structurally characterized by PGC-nanoLC-ESI-MS/MS glycomics. We confirmed that Le^X^ type fucose residues are attached to CEA complex type *N*-glycans and that the global α2-3 sialylation levels were increased on CEA *N*-glycans in the more aggressive gastric carcinoma cells. In addition, and in order to evaluate the prognostic potential of CEA specific glycosylation, we assessed the expression of both CEA and SLe^x^ directly in gastric cancer tissues and evaluated this CEA-SLe^x^ conjoint signature with the respective clinicopathological characteristics. The presence of CEA and SLe^x^ were respectively 98.0% and 86.3% of the total cases with no expression in the normal adjacent tissue. Moreover, PLA assay confirmed the conjoint expression of these two antigens in 74.5% of the analyzed gastric carcinoma cases and was significantly associated with advanced stages gastric carcinomas (stage III+IV) and showed a strong tendency with infiltrative pattern of tumor growth (Ming classification), the presence of venous invasion and patients' poor overall survival. Overall, our results demonstrate that CEA is the major carrier of SLe^x^ antigens in aggressive gastric carcinomas and highlights the combination of CEA and SLe^x^ for potential clinical applications in gastric cancer prognosis. The current serological CEA test has been largely applied in the gastrointestinal oncologic setting, mostly for disease monitoring due to the low specificity for diagnostic purposes 51. By adding protein specific glycosylation as an additional dimension to the serum CEA levels, such a conjoint serological assay, can overcome current limitations and improve the prognostic and diagnostic accuracy. In the future, and according to already described methodologies 52, we envision the use of PLA derived approaches for detection of CEA specific glycoform in blood derived samples from patients.

Our finding opens a window of opportunities for the design of new studies to identify the role of CEA altered glycosylation in tumor cell invasion and metastasis and ultimately the design of new effective therapeutic strategies. Currently, there is a growing evidence regarding the potential role of CEA in cancer cell behavior and metastasis. Several studies have reported its ability in promoting cancer cell metastization through the activation of signaling pathways by the interaction with a specific receptor, hnRNPM4, in specialized cells 53, 54. For instance, it has been described that liver Kupffer cells and lung alveolar macrophages can bind circulating CEA through this specialized receptor, leading to its activation with consequent induction of cytokine release 55 and tyrosine phosphorylation of proteins 56 promoting an inflammatory environment that facilitates tumor cells homing and colony formation. Interestingly, most of the tumors that express CEA have as primary sites for distant metastasis the liver and lung. Furthermore, it was also demonstrated that CEA can bind and activate endothelial cells promoting signaling pathway involved in adhesion, invasion and tumor angiogenesis 57-59. Since CEA have been demonstrated as a very promising molecular target in cancer, several clinical trials, mostly using viral vector vaccines, have been developed 60-64. Interestingly, CEA-specific immune responses have been observed in all these studies with approximately 40% objective clinical responses observed in the treated patients. Recently, chimeric antigen receptor T (CAR-T) therapy targeting CEA has been explored and promising results have been observed 65, 66.

In addition to all these information, SLe^x^, expressed by immune cells, is a well stablished ligand of selectins present in the cell surface of endothelial cells and the main player in rolling, adhesion and extravasation of these cells during the inflammatory process 11. SLe^x^ expression in numerous cancers types its claimed to mimic this process favoring tumor cell invasion and metastization 67. In light of all this knowledge, our findings certainly set the ground for new studies in order to uncover the specific role of this CEA cancer specific glycoform and to design effective therapies in order to reduce cancer associated mortality.

## Conclusion

This present study demonstrates for the first time that SLe^x^ is a terminating saccharide antigen determinant present on CEA *N*-glycans in gastric carcinoma cells. Additionally, the co-existence of CEA+SLe^x^ in gastric carcinoma tissues and its association with the presence of venous invasion and worse patients' overall survival support the biological role of this glycoconjugate in the aggressive behavior of this tumor. Our results set the ground for the combined use of CEA and its altered glycosylation signatures as cancer prognostic marker.

## Supplementary Material

Supplementary figures and tables.

## Figures and Tables

**Table 1:** CEA and SLe^X^ expression in gastric carcinoma tissues and association with clinicopathological data.

	CEA					SLe^X^				
	Neg.	5-25%	25-75%	>75%	P*	Neg.	5-25%	25-75%	>75%	P*
**Laurén classification**										
Intestinal (n=22)	1 (4.5%)	3 (13.6%)	4 (18.2%)	14 (63.6%)	0.289	4 (18.2%)	4 (18.2%)	11 (50%)	3 (13.6%)	0.232
Diffuse (n=5)	0 (0.0%)	0 (0.0%)	3 (60.0%)	2 (40.0%)		0 (0.0%)	0 (0.0%)	4 (80.0%)	1 (20.0%)	
Unclassified (n=24)	0 (0.0%)	1 (4.2%)	10 (41.7%)	13 (54.2%)		3 (12.5%)	4 (16.7%)	8 (33.3%)	9 (37.5%)	
**Carneiro classification**										
Glandular (n=22)	1 (4.5%)	3 (13.6%)	4 (18.2%)	14 (63.6%)	0.481	4 (18.2%)	4 (18.2%)	11 (50%)	3 (13.6%)	0.476
Mixed (n=24)	0 (0.0%)	1 (4.2%)	10 (41.7%)	13 (54.2%)		3 (12.5%)	4 (16.7%)	8 (33.3%)	9 (37.5%)	
Isolated cells (n=4)	0 (0.0%)	0 (0.0%)	2 (50.0%)	2 (50.0%)		0 (0.0%)	0 (0.0%)	3 (75.0%)	1 (25.0%)	
Solid (n=1)	0 (0.0%)	0 (0.0%)	1 (100%)	0 (0.0%)		0 (0.0%)	0 (0.0%)	1 (100%)	0 (0.0%)	
**Ming classification**										
Expansive (n=18)	1 (5.6%)	2 (11.1%)	6 (33.3%)	9 (50%)	0.344	5 (27.8%)	6 (33.3%)	5 (27.8%)	2 (11.1%)	0.008
Infiltrative (n=31)	0 (0.0%)	2 (6.5%)	9 (29.0%)	20 (64.5%)		2 (6.5%)	1 (3.2%)	17 (54.8%)	11 (35.5%)	
Unclassified (n=2)	0 (0.0%)	0 (0.0%)	2 (100%)	0 (0.0%)		0 (0.0%)	1 (50.0%)	1 (50.0%)	0 (0.0%)	
**Lymphatic invasion**										
Absent (n=12)	0 (0.0%)	2 (16.7%)	5 (41.7%)	5 (41.7%)	0.419	3 (25.0%)	0 (0.0%)	6 (50.0%)	3 (25.0%)	0.130
Present (n=39)	1 (2.6%)	2 (5.1%)	12 (30.8%)	24 (61.5%)		4 (10.3%)	8 (20.5%)	17 (43.6%)	10 (25.6%)	
**Venous invasion**										
Absent (n=21)	0 (0.0%)	4 (19.0%)	9 (42.9%)	8 (38.1%)	0.022	4 (19.0%)	4 (19.0%)	9 (42.9%)	4 (19.0%)	0.656
Present (n=30)	1 (3.3%)	0 (0.0%)	8 (26.7%)	21 (70.0%)		3 (10.0%)	4 (13.3%)	14 (46.7%)	9 (30.0%)	
**pTNM**										
I+II (n=30)	1 (3.3%)	3 (10.0%)	12 (40.0%)	14 (46.7%)	0.329	6 (20.0%)	4 (13.3%)	13 (43.3%)	7 (23.3%)	0.470
III+IV (n=21)	0 (0.0%)	1 (4.8%)	5 (23.8%)	15 (71.4%)		1 (4.8%)	4 (19.0%)	10 (47.6%)	6 (28.6%)	
***Total***	**1 (2.0%)**	**4 (7.8%)**	**17 (33.3%)**	**29 (56.9%)**		**7 (13.7%)**	**8 (15.7%)**	**23 (45.1%)**	**13 (25.5%)**	
**Overall Survival**										
Average survival (months±SD)	ND	ND	67.6 ± 12.7	58.6 ± 8.5	0.117	76.8 ± 19.3	78.2 ± 16.2	51.7 ± 10.6	82.3 ± 11.0	0.293
95% CI	ND	ND	42.5 - 92.7	41.8 - 75.4		38.9 - 11.7	46.5 - 110.0	30.9 - 72.4	60.6 - 104.0	

**Table 2:** Detection of CEA+SLe^X^ by PLA analysis and correlation with clinicopathological data.

	PLA signal		
	Neg.	Pos.	P*
**Laurén classification**			
Intestinal (n=22)	6 (27.3%)	16 (72.7%)	0.942
Diffuse (n=5)	1 (20.0%)	4 (80.0%)	
Unclassified (n=24)	6 (25.0%)	18 (75.0%)	
**Carneiro classification**			
Glandular (n=22)	6 (27.3%)	16 (72.7%)	0.357
Mixed (n=24)	5 (20.8%)	19 (79.2%)	
Isolated cells (n=4)	1 (25.0%)	3 (75.0%)	
Solid (n=1)	1 (100%)	0 (0.0%)	
**Ming classification**			
Expansive (n=18)	8 (44.4%)	10 (55.6%)	0.063
Infiltrative (n=31)	5 (16.1%)	26 (83.9%)	
Unclassified (n=2)	0 (0%)	2 (100%)	
**Lymphatic invasion**			
Absent (n=12)	5 (41.7%)	7 (58.3%)	0.138
Present (n=39)	8 (20.5%)	31 (79.5%)	
**Venous invasion**			
Absent (n=21)	8 (38.1%)	13 (61.9%)	0.081
Present (n=30)	5 (16.7%)	25 (83.3%)	
**pTNM**			
I+II (n=30)	11 (36.7%)	19 (6.3%)	0.028
III+IV (n=21)	2 (9.5%)	19 (90.5%)	
***Total***	13 (25.5%)	38 (74.5%)	
**Overall Survival**			
Average survival (months±SD)	88.1 ± 13.0	60.5 ± 7.8	0.168
95% CI	62.6 - 113.6	45.1 - 75.9	

## Abbreviations

BSA: bovine serum albumin
CA125: cancer antigen 125
CEA, CEACAM5: Carcinoembryonic antigen
CHSJ: Centro Hospitalar São João
DAB: 3,3′-diaminobenzidine tetrahydrochloride
DTT: Ditiotreitol
ECL: enhanced chemiluminescence
GC: Gastric cancer
MALDI-TOF/TOF: Matrix assisted laser desorption/ionisation - time of flight
PAS: periodic acid-Schiff
PBS: phosphate-buffered saline
PBST: phosphate-buffered saline containing 0.05% Tween 20
PGC: porous graphitized carbon
PLA: proximity ligation assay
PMF: Peptide mass fingerprint
PSA: prostate-specific antigen
SDS-PAGE: sodium dodecyl-polyacrylamide gel electrophoresis
SLe^x^: sialyl-Lewis X
ST3Gal IV: α2-3 sialyltransferase IV
