# Supplementary material for: Carcinoembryonic antigen carrying SLeX as a new biomarker of more aggressive gastric carcinomas
Source: Theranostics. 2019 Oct 11;9(24):7431–46. doi: 10.7150/thno.33858 (PMC6831293; doi:10.7150/thno.33858)
Supplement: Supplementary file 1 — Supplementary figures and tables. [file thnov09p7431s1.pdf]

Supplementary Material

Tables

Table S1: SLe^x antigen positive proteins identified from MST3Gal IV cell using MALDI-TOF-MS/MS. The list of peptides obtained from Mascot can be found in the supplementary material.

Spot ID	*Protein description*	*UniProtKB Accession number*	*Number of identified peptides*	*% Sequence coverage*	*Protein score**
1	Carcinoembryonic antigen-related cell adhesion molecule 5	P06731	4	8	92
2	Ras GTPase-activating-like protein IQGAP1	P46940	35	21	268
	Carcinoembryonic antigen-related cell adhesion molecule 5	P06731	5	10	234

* Protein scores greater than 56 are considered significant ($p<0.05$)

Table S2 - List of released *N*-glycans detected on immunoprecipitated CEA (500 ng) from Mock and ST3GalIV cell lines. *N*-glycans were released from immunoprecipitated CEA using PNGase F, analyzed by PGC-nanoLC-ESI-MS/MS and qualitatively and quantitatively assessed. Each *N*-glycan has an identification number (ID), a detected *m/z* (Detected [M-H]$^-$), a theoretical *m/z* (Theor. [M-H]$^-$), a mass error in Da (ΔM), retention time in minutes (RT), proposed structure and relative abundances for CEA derived from Mock and MST3GalIV cells. The monosaccharides are represented following the recommendation of the SNFG [28].

						Relative abundances(%)	
ID	Detected [M-H]$^-$	Theor. [M-H]$^-$	ΔM (Da)	RT (min)	Structure	Mock	MST3GalIV
1	1235.49	1235.44	0.05	25.1	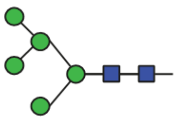	2.139	2.034
2	1397.55	1397.49	0.06	19.1	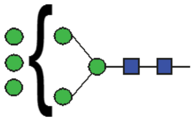	12.902	10.821
3	1559.58	1559.55	0.03	19.1	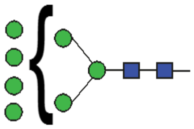	7.593	8.104
4	1559.60	1559.55	0.06	18.0	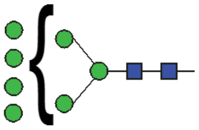	5.445	8.595
5	1721.79	1721.60	0.19	19.0	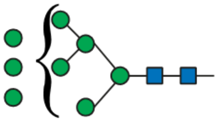	17.020	15.928
6	1883.76	1883.65	0.10	18.0	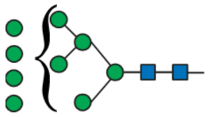	20.185	18.254
7	1875.83	1875.67	0.15	24.3	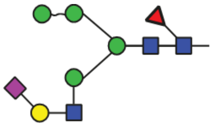	2.761	0.000
8	1875.77	1875.67	0.09	32.9	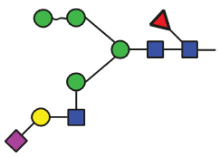	0.000	2.243

9	1891.75	1891.67	0.08	22.6	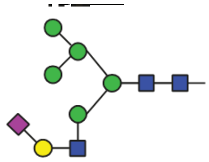	2.817	1.438
10	2078.80	2078.75	0.04	29.1	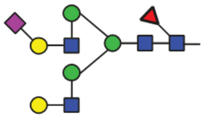	4.934	2.273
11	2224.89	2224.81	0.08	27.5	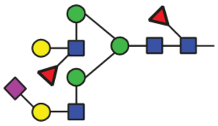	8.866	0.000
12	2281.83	2281.83	0.01	21.1	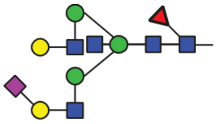	2.893	0.000
13	2281.85	2281.83	0.01	26.9	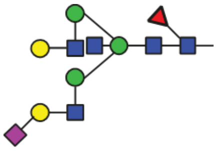	0.000	1.824
14	2572.91	2572.93	0.02	21.6	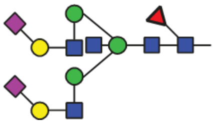	4.272	0.000
15	2572.94	2572.93	0.01	27.9	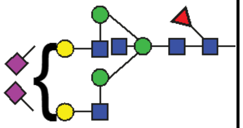	1.687	3.133
16	2572.93	2572.93	0.00	35.4	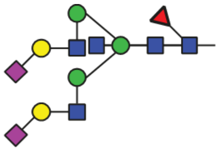	0.000	7.041
17	2369.92	2369.85	0.07	28.6	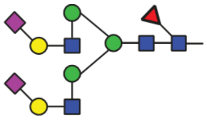	6.485	0.000
18	2369.87	2369.85	0.02	37.1	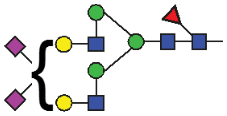	0.000	5.370

19	2369.86	2369.85	0.01	43.4	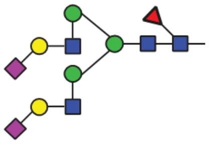	0.000	12.942

Figures

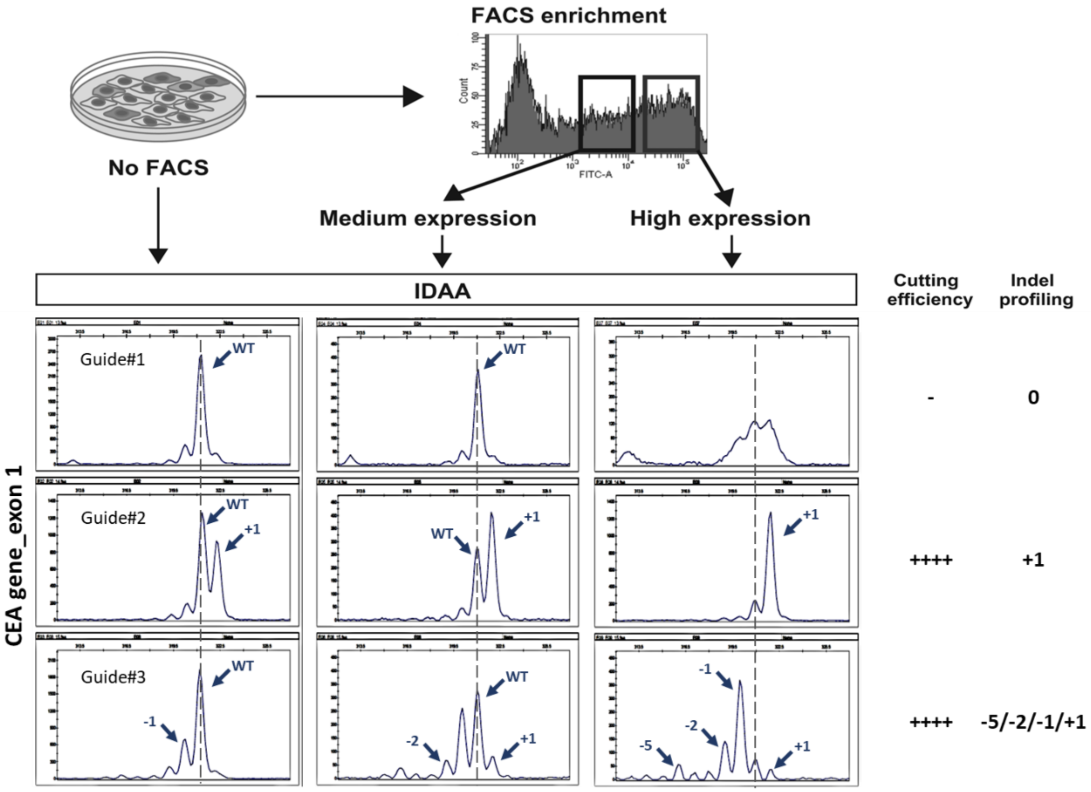

Figure S1 – CEA gRNA selection and validation using FACS enrichment and IDAA monitoring. IDAA profiles for the three different gRNAs designs (Guide #1–3) monitored in the original pool of transfected HEK293T cells and after FACS enrichment for cells with medium or high Cas9-GFP expression. Guide#1 showed no cutting efficiency whereas Guide#2 displayed the highest cutting efficiency with a rather homogenous +1 indel, which was easily detectable even in the original cell pool. Guide#3 displayed lower efficiency compared to Guide#2, detectable after enrichment for GFP expression with heterogeneous indel formation.

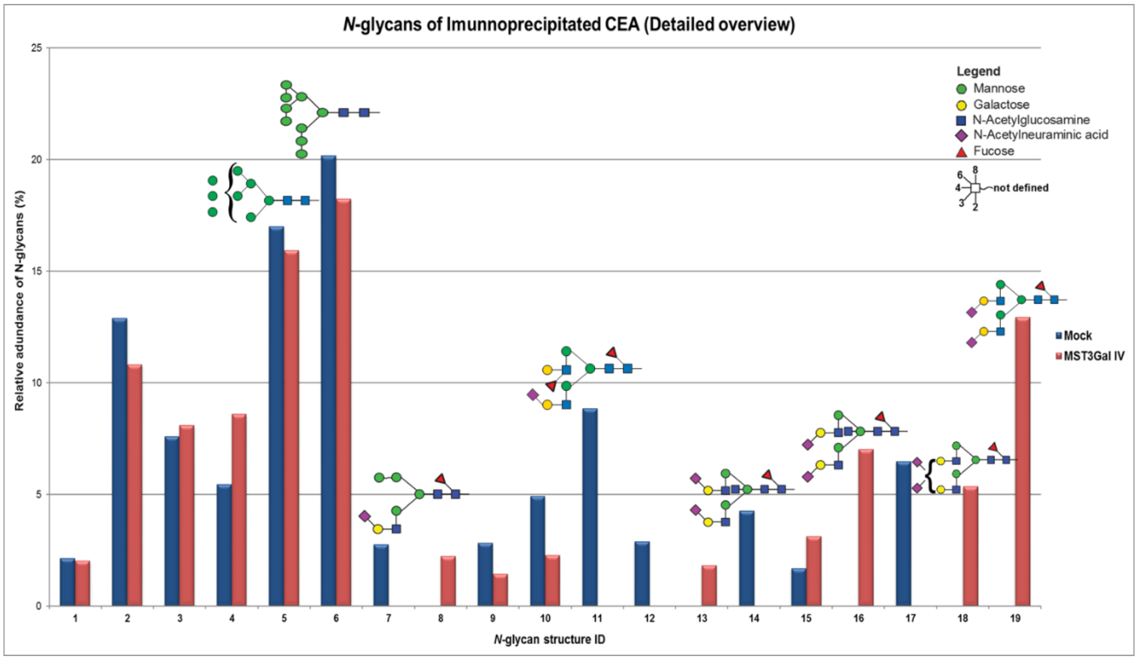

Figure S2 – Overview of *N*-glycans identified on immunoprecipitated CEA from Mock and MST3GalIV cell lines. The *N*-glycan ID numbers were represented along the horizontal axis against the relative abundance (%) of *N*-glycans in the vertical axis. The relative abundances of each individual *N*-glycan of CEA is different in Mock or MST3GalIV cells.
